# The history, biological relevance, and potential applications for polyp bailout in corals

**DOI:** 10.1002/ece3.7740

**Published:** 2021-06-05

**Authors:** Maximilian Schweinsberg, Fabian Gösser, Ralph Tollrian

**Affiliations:** ^1^ Department of Animal Ecology, Evolution and Biodiversity University of Bochum Bochum Germany

**Keywords:** Genet–Ramet system, model system, polyp bailout, scleractinian corals, stress response

## Abstract

Corals have evolved a variety of stress responses to changing conditions, many of which have been the subject of scientific research. However, polyp bailout has not received widespread scientific attention, despite being described more than 80 years ago. Polyp bailout is a drastic response to acute stress in which coral colonies break down, with individual and patches of polyps detaching from the colony and the calcareous skeleton Polyps retain their symbiotic partners, have dispersal ability, and may undergo secondary settlement and calcification. Polyp bailout has been described worldwide in a variety of anthozoan species, especially in Scleractinia. It can be induced by multiple natural stressors, but also artificially. Little is known about the evolutionary and ecological potential and consequences of breaking down modularity, the dispersal ability, and reattachment of polyps resulting from polyp bailout. It has been shown that polyp bailout can be used as a model system, with promise for implementation in various research topics. To date, there has been no compilation of knowledge on polyp bailout, which prompted us to review this interesting stress response and provide a basis to discuss research topics and priorities for the future.

## Graphical abstract

The present review addresses the stress response polyp bailout, including the current state of research and theoretical aspects of the evolutionary and ecological consequences. Furthermore, the review deals with the possible use of polyp bailout in future research.

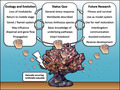

## 1 INTRODUCTION

### 1.1 Plasticity of life‐history strategies in modular invertebrates

Scleractinian corals (Cnidaria, Anthozoa, Scleractinia), a group severely affected by climate change and biodiversity loss, are mostly modular organisms with a broad diversity of life‐history strategies (Baird et al., 2009; Bridge et al., 2020; Carlon, 1999; Hidaka, 2016). Modularity is a widespread phenomenon in sessile, marine invertebrates within the phyla Porifera, Bryozoa, Cnidaria, Entoprocta, Hemichordata, and Urochordata (Dyrynda, 1986; Hughes, 1989; Jackson & Coates, 1986; Rinkevich, 2002; Rosen, 1986). Modular organisms are characterized by flexible developmental programs including clonal and modular growth, the ambiguity of senescence, diverse reproductive strategies within an organism, and different growth forms.

In particular, the repeated building blocks that form modular organisms result in extensive morphological plasticity and flexibility (Hageman, 2003; Harvell, 1991; Hughes, 2005; Marfenin, 1997), as well as high variability in the development of life‐history strategies (Bridge et al., 2020; Hall & Hughes, 1996; Hiebert et al., 2020; Jackson & Coates, 1986). These building blocks are often repeating organizational units (e.g., polyps) that can be regarded as morphological individuals. However, the entirety of the building blocks of a modular organism (colony) can also be considered as a distinct physiological individual (Hageman, 2003). Polyps within a colony typically have integrated physiologies, the growth form of the colony is coordinated, metabolites are translocated between polyps, and some parts of the colony are often specialized for reproduction or defense (Hughes et al., 1992). Finally, all polyps and colonies descended from the same zygote have the same genotype and belong to a genetic individual called a genet. Physically separate but genetically identical modules of a genet are referred to as ramets (Baums et al., 2006; Harper, 1977; Heyward & Collins, 1985). A genet may persist as an intact unit throughout life or produce ramets through various (asexual) processes (Bastidas et al., 2004; Baums et al., 2006; Highsmith, 1982; Stoddart, 1983).

### 1.2 Stress and responses of corals

Due to their sessile lifestyle and the associated inability to physically escape harsh conditions, corals have evolved various life‐history traits concerning reproduction, growth, and competition that facilitate survival and mitigate stressful conditions (Apprill, 2020; Lesser, 1997; Sammarco, 1982; Weber et al., 2006). However, there is reasonable doubt that the adaptive potential of corals will be sufficient to compensate for climate change with most populations already living near to their thermal and physiological limits (Epstein et al., 2019; Hughes et al., 2017; van Oppen et al., 2017). Therefore, their survival depends on a number of factors: the threshold of tolerable conditions, their ability to survive near the range limit, their capacity to regenerate after damage, and the development of various stress responses to cope with unfavorable biotic and abiotic factors (Birkeland, 1997; Harrison & Booth, 2007; Mumby & Steneck, 2008).

Numerous environmental conditions have been identified as detrimental for coral colonies resulting in the formation of stress responses; these include higher than usual sea surface temperatures (Hoegh‐Guldberg, 1999; Hughes et al., 2017; Lesser, 1997), sedimentation (Anthony & Larcombe, 2000; Weber et al., 2006; Wiedenmann et al., 2013), shifts in nutrient availability (Morris et al., 2019; Wiedenmann et al., 2013), pollution (Negri et al., 2012), ocean acidification (Hoegh‐Guldberg et al., 2007), and epizootics (Harvell et al., 2007). These stressors affect almost all life stages of scleractinian corals, resulting in a great variety of adaptations in coral life‐history traits with associated plasticity of responses. These stress responses range from physiological changes (Poli et al., 2017; Putnam et al., 2016; Ricardo et al., 2016) and a sexual‐to‐asexual reproductive switch (Ayre & Resing, 1986; Harrison, 2011; Highsmith, 1982; Sammarco, 1982), to drastic responses such as coral bleaching (Lesser, 1997; Stuart‐Smith et al., 2018). Typically, stress responses such as coral bleaching seem to occur during long‐term or chronic stressor exposure, whereas a remarkably different stress reaction, the polyp bailout, is detectable during intense, acute stress over a short time frame (Chuang & Mitarai, 2020).

Polyp bailout has only recently returned to the scientific focus, despite being described more than 80 years ago by Goreau and Goreau (1959) or even earlier by Kawaguti (1942). This remarkable response enables corals to break down the colonial integrity by degrading connective tissue and releasing polyps from the calcareous skeleton. By doing so, polyp bailout creates new ramets with dispersal potential, providing a promising acute survival mechanism for scleractinian corals exposed to severe stress conditions. The increasing number of reports about polyp bailout in recent years suggests that the phenomenon is more widespread in scleractinian corals than previously thought. This has prompted us to compile and standardize the previous information to create a basis for future research. This review aims to give the first scientific overview of the state of knowledge on polyp bailout, to highlight possible ecological and evolutionary potential and consequences, and to summarize expected research topics and priorities for the future.

## 2 OVERVIEW OF POLYP BAILOUT IN ANTHOZOA

### 2.1 Physiological processes of polyp bailout

**FIGURE 1:**
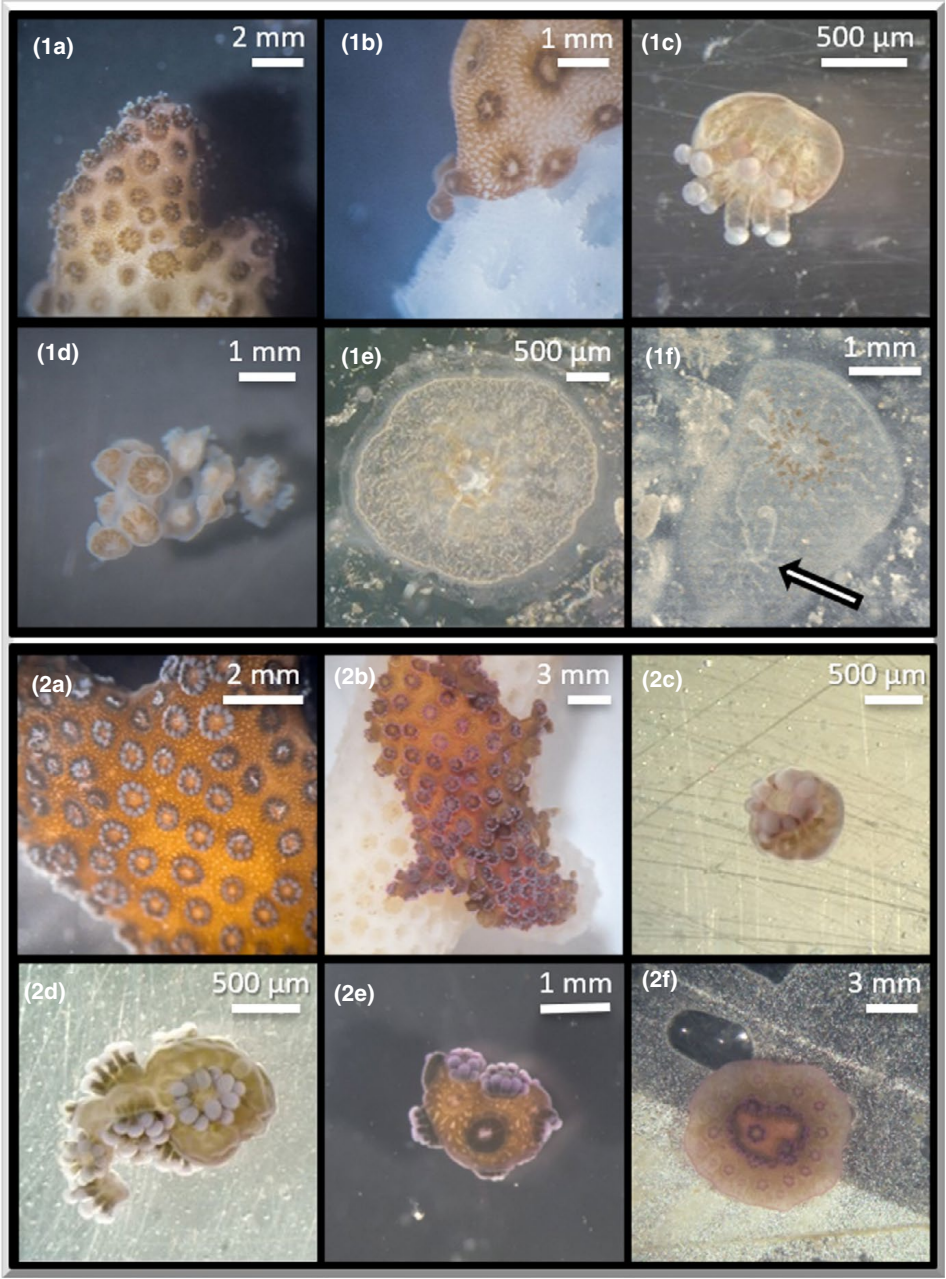
Sequence of polyp bailout with direct resettlement exemplarily in *Pocillopora acuta* (1a–f) and delayed resettlement exemplarily in *Stylophora pistillata* (2a–f). Both species are able of direct as well as delayed settlement. Healthy *P. acuta* (1a). Polyps detach from the colony during polyp bailout (1b). Polyps detach from *P. acuta*, either as single polyps (1c) or as patches (1d). Single polyp settled on an object slide within 24 hr (1e). After two weeks, the formation of a calcareous skeleton and the budding of a secondary polyp in *P. acuta* could be observed (1f, arrow). Healthy *S. pistillata* (2a). Polyps detach from *S. pistillata* (2b), either as single polyps (2c) or as patches (2d). Free‐floating polyp (2e) one month after polyp bailout with renewed skeletogenesis (2e). After two months, reattachment to the surface and budding of new polyps had occurred (2f)

In Anthozoa, particularly scleractinian corals, the typical growth of a colony begins with settled planula larvae developing into a primary polyp that divides clonally by budding (Hughes, 1983). Continuous division of the polyps results in a colony consisting of hundreds or thousands of single modules. Within this colony, the individual polyps remain connected to each other by a connective tissue called the coenosarc (Hall & Hughes, 1996). During polyp bailout, this sessile, colonial unit is broken up. First, the tentacles of polyps undergoing bailout retract, followed by degradation of the coenosarc beginning at the corallite wall. Individual polyps then retract into their respective corallite and lastly detach themselves completely from the skeleton. They retain their dinoflagellate symbionts (family: Symbiodiniaceae), can reattach themselves to a surface, and are able to grow into a new colony (Sammarco, 1982; Wecker et al., 2018; see Figure 1).

### 2.2 First observations of polyp bailout were incidental

The first published incidental observation of polyp bailout was in 1942; Kawaguti described a "pseudo‐planula” as a colony of *Acropora tenuis* began deteriorating while being kept in an aquarium. The coenosarc broke down, bare skeleton was visible, but the polyps remained alive. Later, a freshly formed coral calice appeared attached to the wall of the aquarium complete with a healthy coral polyp. The author believed that the polyp was released from the coral colony and coined the term “pseudo‐planula,” but described its development as different from that of larvae (Kawaguti, 1942). A similar incidental observation was made by Goreau and Goreau when they reported that polyps of starving corals (species not documented) were able to detach themselves completely from the corallite. The polyps stayed alive for several weeks, although without showing any evidence of renewed skeletogenesis (Goreau & Goreau, 1959).

### 2.3 Scientific description of polyp bailout

The first intentional scientific description of the process, induced by a lack of water flow, was by Sammarco in 1982, who coined the term “polyp bailout.” He observed polyps of *Seriatopora hystrix* detaching from the colony after the coenosarc dissolved. Sammarco documented polyps’ survivorship and their potential to reattach to the substrate and grow into a new colony. Polyps retained their symbiotic algae, could be passively transported by water movement, attached to the substrate, and secreted a skeleton (Sammarco, 1982). A lack of water flow also induced polyp bailout in colonies of *Plesiastrea versipora* (Ritchie et al., 1997). In these colonies, the coenosarc progressively thinned and contracted toward the calyces until the polyps were completely detached from each other, as described by Sammarco (1982). The resulting polyps survived in glass dishes in the laboratory for three weeks or more but did not secrete a skeleton.

### 2.4 Stressors known to induce polyp bailout

In the last two decades, polyp bailout has been scientifically described in at least ten scleractinian corals, in three alcyonacean soft corals, and one antipatharian coral (see Table 1). In most of the experiments, polyp bailout was induced by short‐term extreme stress exposure.

**TABLE 1:** Reported cases of coral species undergoing polyp bailout including information about stressors, geography, and author

Coral species	Order	Stressor	Geographic region of origin	Author (year)
*Acropora tenuis*	Scleractinia	N.A.	Pacific Ocean, Japan	Kawaguti (1942)
		DCMU	Pacific Ocean, Japan	Yuyama et al. (2012)
		DCMU	Pacific Ocean, Japan	Kariyazono and Hatta (2015)
*Astroides calycularis*		Lack of food	Mediterranean Sea, Spain	Serrano et al. (2018)
*Cladocora caespitosa*		Heat	Mediterranean Sea, Croatia	Kruzic (2007)
*Oculina patagonica*		pH	Red Sea, Israel	Kvitt et al. (2015)
*Plesiastrea versipora*		Rearing conditions	Pacific Ocean, Australia	Ritchie et al. (1997)
*Pocillopora acuta*		Salinity	Pacific Ocean, Japan	Chuang and Mitarai (2020)
		Salinity	Pacific Ocean, Japan	Chuang et al. (2021)
		Ca‐free seawater	Pacific Ocean, China	Luo et al. (2020)
		Ca‐free seawater	Pacific Ocean, China	Pang et al. (2020)
*Pocillopora damicornis*		N.A.	Pacific Ocean, Costa Rica	Wild et al. (2014)
		Abrasion by macroalgae	Pacific Ocean, Singapore	Lee et al. (2012)
		pH	Red Sea, Israel	Kvitt et al. (2015)
		Salinity	Red Sea, Israel	Shapiro et al. (2016)
		Heat	Great Barrier Reef, Australia	Fordyce et al. (2017)
		Chlordecone	Pacific Ocean, French Polynesia	Wecker et al. (2018)
		Salinity	Pacific Ocean, China	Liu et al. (2020)
*Seriatopora hystrix*		Rearing conditions	Great Barrier Reef, Australia	Sammarco (1982)
		Salinity	Red Sea, Israel	Shapiro et al. (2016)
*Stylophora pistillata*		Salinity	Red Sea, Israel	Shapiro et al. (2016)
*Tubastraea coccinea*		Rearing conditions	Atlantic Ocean, Brazil	Capel et al. (2014)
*Acanthogorgia armata*	Alcyonacea	Rearing conditions	Atlantic Ocean, Azores	Rakka et al. (2019)
*Acanella arbuscula*		Rearing conditions	Atlantic Ocean, Azores	Rakka et al. (2019)
*Eunicea flexuosa*		Aerial exposure	Atlantic Ocean, US Virgin Islands	Wells and Tonra (2021)
*Antipathella subpinnata*	Antipatharia	Rearing conditions	Mediterranean Sea, Italy	Coppari et al. (2020)

Abbreviation: N.A., not available.

Severe temperature changes induced polyp bailout in two different coral species. A temperature increase of 4°C higher than the normal maximum summer value of 26°C in *Cladocora caespitosa* colonies led to bailout of the polyps, which were later observed attached to the glass of the aquaria (Kruzic, 2007). Similar processes were observed in a study that aimed to simulate a bleaching event in *P. damicornis*, which led to the onset of polyp bailout (Fordyce et al., 2017). Further experimental studies have shown that polyp bailout is inducible under reduced pH conditions (pH 7.2) in the two corals *Oculina patagonica and P. damicornis* (Kvitt et al., 2015). Osmoregulatory stress (increase in salinity of >15‰) has been shown to induce polyp bailout in three different pocilloporid coral genera (Chuang et al., 2021; Chuang & Mitarai, 2020; Liu et al., 2020; Shapiro et al., 2016). Two other recent studies used calcium‐free seawater as an artificial stressor to induce bailout in pocilloporid corals (Luo et al., 2020; Pang et al., 2020).

Herbicides and insecticides have been shown to induce the polyp bailout stress response in two coral genera. Primary polyps of *Acropora tenuis* exposed to treatments with varying concentrations of 3′‐(3,4‐dichlorophenyl)‐1,1‐dimethylurea (DCMU, or Diuron) showed polyp bailout (Kariyazono & Hatta, 2015; Yuyama et al., 2012). Concentrations higher than 30 µg/L of the insecticide chlordecone also induced polyp bailout in *Pocillopora damicornis* (Wecker et al., 2018). Furthermore, abrasion and secondary metabolites from algae led to the bailout of freshly settled *P. damicornis* larvae (Lee et al., 2012).

In an interaction study between the model coral pathogen *Vibrio coralliilyticus* and its coral host *P*. *damicornis*, the authors described resorption of the coenosarc and finally polyp bailout in infected fragments (Gavish et al., 2018). Low planktonic food availability most likely induced the dissolution of the colonial unity and consequently polyp bailout in *Astroides calycularis* (Serrano et al., 2018). Stressed polyps of *Tubastraea coccinea* were able to detach without skeletal parts, and after some time, one of the polyps secreted a new skeleton and reattached to the surface (Capel et al., 2014). Wild et al. (2014) described a process similar to polyp bailout in *Pocillopora damicornis* colonies in Costa Rica. However, the authors found neither visible signs of infection nor abiotic or biotic factors causing polyp detachment.

In addition to the increasing number of studies for polyp bailout in Scleractinia (Hexacorallia), several descriptions in Anthozoa have been published in the last two years. The cold‐water corals *Acanthagorgia armata* and *Acanella arbuscula*, both alcyonacean species collected in the Azores, were kept in aquaria and showed polyp bailout in the tanks (Rakka et al., 2019). Polyp bailout was further described in another alcyonacean species, *Eunicea flexuosa*. Here, aerial exposure triggered polyp bailout in the colonies (Wells & Tonra, 2021). Additionally, evidence for the occurrence of polyp bailout in the species *Antipathella subpinnata* (Antipatharia) under aquaria conditions has been described (Coppari et al., 2020).

### 2.5 Signaling pathways during polyp bailout

Although the regulatory machinery of polyp bailout is far from being understood, multiple studies have started to reveal the underlying signaling pathways that take place during the stress response in pocilloporid corals. Studies concerning this section of polyp bailout have shown to date that similar signaling pathways are detectable during pH‐, chemical‐, and salinity‐induced stress responses (Chuang & Mitarai, 2020; Kvitt et al., 2015; Wecker et al., 2018). In the two corals *Oculina patagonica* and *Pocillopora damicornis*, tissue‐specific apoptosis was responsible for the dissolution of the coenosarc. Using a protein activity assay, this link between tissue‐specific apoptosis and polyp bailout was established for the first time. In addition, it was shown that high caspase activity was most likely responsible for the apoptotic processes leading to coenosarc dissolution (Kvitt et al., 2015). Wecker et al. (2018) performed a transcriptomic stress study with *P. damicornis*, which found overexpression of transcripts involved in apoptosis and degradation of cellular matrix proteins, supporting the assumptions of Kvitt et al. (2015). Furthermore, this study suggested that proteolytic enzymes, for example, cathepsins, affect the extracellular matrix between polyps and the calcified skeleton, promoting polyp detachment by proteolysis (Wecker et al., 2018). Based on these previous findings, a study used hypersalinity to induce polyp bailout in *P. acuta* (Chuang & Mitarai, 2020). The authors found that *P. acuta* showed significant activation of tumor necrosis factor signaling and fibroblast growth factors in addition to upregulation of apoptosis and proteolysis, leading to polyp survival. This study was also the first to question the relevance of interkingdom communication between microbes, algae, and the eukaryotic coral host during the polyp bailout response (Chuang & Mitarai, 2020). In a subsequent study, again using hypersalinity stress, a return to pre‐bailout expression patterns was reported in detached regenerating polyps (Chuang et al., 2021).

In summary, polyp bailout has been reported for anthozoans in general and scleractinian corals in particular. It was observed in tropical, temperate, and cold‐water species (Capel et al., 2014; Kruzic, 2007; Rakka et al., 2019; Sammarco, 1982), over a wide geographic range (e.g., Red Sea, Indo‐Pacific, Mediterranean Sea, Atlantic ocean, Caribbean and Pacific Coast, Great Barrier Reef) and for a variety of stressors (see Table 1). It was also present in zooxanthellate species, as well as in azooxanthellate species. This implies that the process of polyp bailout might be a common stress response, at least in three orders of the class of Anthozoa. By restricting our literature search strictly to the phrase “polyp bailout,” it is likely that more species are capable of the process, but it has been named differently or possibly categorized as asexual reproduction, for example, polyp fission or polyp balls in zoantharians (González‐Muñoz et al., 2019) or even in scleractinians like *Euphyllia* sp. (Bornemann, 2006).

## 3 IS POLYP BAILOUT DISTINCT FROM ASEXUAL REPRODUCTION PROCESSES?

In cnidarians, the borders of sexual and asexual reproduction are rather faint; research continuously adds new insights into these reproductive processes. Asexual reproduction is a field of enormous variation among and even within species, and numerous different forms of asexual reproduction, from mechanical fragmentation to reverse development, to propagation, have been described in cnidarians (reviewed in Fautin, 2002; Piraino et al., 2004). In fact, most Anthozoa possess multiple reproductive modes, such as scissiparity and budding, consistent with the high degree of developmental plasticity of modular animals (Bocharova & Kozevich, 2011; Edwards & Moore, 2009; Fautin, 2002; Harrison, 2011; Hughes, 1983; Ohdera et al., 2018; Orejas et al., 2002).

Colony break‐up is a common response of cnidarians to a variety of adverse conditions (Babcock, 1991; Edmunds & Elahi, 2007; Elahi & Edmunds, 2007). Indeed, there are several processes described in cnidarians returning to a mobile individual stage (Piraino et al., 2004), leading to the question: Can polyp bailout be differentiated or distinguished from other similar developmental aspects such as asexual reproduction and propagation mechanisms and reverse developmental processes (Piraino et al., 2004)? In particular, where and how to place polyp bailout in a profound categorization between asexual modes of reproduction or reverse developmental processes is currently proving difficult.

Polyp bailout appears to occur in various ways in anthozoans and especially in scleractinians, but is triggered by detrimental conditions (Acosta et al., 2005; Rakka et al., 2019; Sammarco, 1982). Sammarco described polyp bailout as a stress response as early as 1982 but was also the first to question whether polyp bailout is a possible mode of asexual reproduction or consecutively of propagation. Piraino et al. (2004) went even further by assuming that polyp bailout could be a further step toward reactivation of early developmental programs in Anthozoa and boundaries of asexual processes and their outcomes should be considered faint. Therefore, we will hereafter restrict the term asexual reproduction to processes that result in the production of new independent modules that form physically separate but genetically identical clones (ramets) of the same genotype lineage (genet) (Harrison, 2011; Highsmith, 1982; Richmond, 1997), in contrast to budding, the iterative addition of modules, like polyps, that lead to the growth of colonies, although the physiological and cellular mechanisms may be similar (Hughes, 1983). In this context, the ramet can be seen as the ecological individual and the genet as the evolutionary individual (Lasker & Coffroth, 1999).

### 3.1 Asexual reproductive processes

Modes of asexual reproduction in anthozoans are numerous and diverse. It can include fragmentation, the breaking off of skeletal parts, especially in species with fine branches or thin plates, by physical impact or other damage, with subsequent attachment of these parts to substrate (Baums et al., 2006; Highsmith, 1982). Further modes are colony fission, longitudinal and transverse division of colonies by dividing the tissue into smaller modules (Bastidas et al., 2004), formation of polyp balls as described in some *Goniopora* colonies (Rosen & Taylor, 1969), and formation of polyps from an anthocaulus by regenerating damaged tissues in fungiids (Kramarsky‐Winter & Loya, 1996). Asexual planula larvae have been described for *P. damicornis* and *P. acuta* (Ayre & Miller, 2004; Oury et al., 2019; Stoddart, 1983), *Tubastraea coccinea*, *Tubastraea diaphana*, *Tubastraea tagusensis* (Ayre & Resing, 1986; Capel et al., 2017), and *Oulastrea crispata* (Lam, 2000). However, to our knowledge, it is not yet clear which process leads to asexually brooded planulae: parthenogenesis, budding, or self‐fertilization.

### 3.2 Similar stress responses in other cnidarians

It is striking that some mechanisms of asexual reproduction resemble those of polyp bailout. For example, processes similar to polyp bailout, in which polyps are ejected from corallites, have also been described in other Scleractinia (Kramarsky‐Winter et al., 1997; Rosen & Taylor, 1969). Unlike polyp bailout, in which detached polyps do not have skeletal remains, these polyps still retained parts of their skeleton. Asexual processes, that are similar to polyp bailout, have also been described in other anthozoans, such as zoanthids (Acosta et al., 2005), although this response does not appear to be stress‐induced, unlike polyp bailout, which has only been described under deleterious conditions (Fordyce et al., 2017; Kvitt et al., 2015; Sammarco, 1982). A counterpart to polyp bailout is apparently also found in hydroids. Here, hydranths can be released, become pelagic, and are able to resettle and differentiate (Gravier‐Bonnet, 1992). This process could also be a mechanism of reverse development. Processes of reverse development have been reported in several hydrozoans (Bavestrello et al., 2000; Piraino et al., 2004), scyphozoans (Laurie‐Lesh & Corriel, 1973), and anthozoans (Pearse, 2002; Piraino et al., 2004). Other processes may include development into dormant stages with greatly reduced metabolic functions (Piraino et al., 2004) and reverse development of adults to earlier developmental stages (Jackson & Coates, 1986). In Scleractinia, larvae of *P. damicornis* that had already settled and transformed into a primary polyp have been observed to undergo “reversible metamorphosis” by leaving their corallites and regressing to a planula‐like stage (Richmond, 1985; Te, 1992). This opens the possibility of finding more favorable sites for settlement and metamorphosis, which in turn hypothetically leads to higher survival rates.

### 3.3 Modern methods could shed light on processes of polyp bailout

Further research is needed to distinguish between polyp bailout and reverse development or to prove their similarity. Modern microscopic methods can help to further categorize polyp bailout. For example, the extent to which detached polyps retain their tissue structures or undergo fundamental restructuring is unknown. Scanning electron microscopy (SEM) and transmission electron microscopy (TEM) can provide insights if detached polyps differ from healthy polyps or multicellular ball‐like aggregates formed in primary cultures of *P. damicornis* cells (Lecointe et al., 2013). For polyps detached by salinity, tissue clearing (TC) coupled with light sheet fluorescence microscopy (LSFM) has shown the first recordings of apoptosis and proliferation at the single‐cell level (Liu et al., 2020). Next‐generation sequencing techniques will also contribute to a better understanding of polyp bailout (Chuang et al., 2021; Chuang & Mitarai, 2020; Wecker et al., 2018). For example, Chuang et al. (2021) demonstrated the return of detached polyps to physiological integrity with concomitant normalization of mRNA expression.

Interestingly, the current boundaries between polyp bailout, asexual reproductive processes, and reproduction are blurred. Although it has been suggested that asexual reproductive processes are more important and even increase under unfavorable conditions (Fautin, 2002; Honnay & Bossuyt, 2005; Richmond, 1997), others argue that asexual reproduction dominates in stable environments and takes advantage of locally adapted genotypes (Miller & Ayre, 2004).

Although there is an overlap with asexual reproduction, published work to date indicates that polyp bailout is a stress response. Previous studies suggest that parts of the processes during polyp bailout bear similarities to those of reverse development mechanisms that are described in stressful situations in corals (Chuang & Mitarai, 2020; Kvitt et al., 2015). During polyp bailout, polyps detach from the skeleton; therefore, the connection of the tissue with the skeleton must be dissolved. Furthermore, the detached polyps retract their tentacles and assume a spherical shape shortly after leaving the skeleton. The following reattachment to the substrate by flattening of the polyp base, extension of the tentacles, and resumption of skeletogenesis all imply a partial rearrangement of the running genetic cascade during polyp bailout. This assumption is supported by recent studies from Kvitt et al. (2015), Wecker et al. (2018), and Chuang et al. (2021). Studies on polyp bailout have shown signaling pathways similar to those described in other coral stress/immune responses, for example, programmed cell death (Chuang & Mitarai, 2020; Palmer, 2018). Interestingly, colony break‐up is precisely this aspect of transitioning from a colonial, sessile phase to a mobile state and thus escaping from acute stress conditions, which is the great contrast to other stress responses within corals. However, we summarize that polyp bailout is a stress response that additively utilizes established life‐history traits similar to asexual reproduction in anthozoans.

## 4 POTENTIAL SELECTIVE CONSEQUENCES

Many questions remain regarding the differentiation of the underlying genetic cascade to polyp bailout from that of reverse development or asexual reproductive processes. The literature clearly states that polyp bailout is an established stress response in a variety of different corals, independent of spatial and temporal circumstances. In the course of our literature search, especially with reference to older literature, we encountered a number of publications describing stress responses or asexual reproductive processes that could be potential polyp bailout responses (Jackson & Hughes, 1985; Kruzic, 2007; Rosen & Taylor, 1969; Wild et al., 2014). Hence, polyp bailout can be considered a ubiquitous and ecologically relevant stress response, which suggests that polyp bailout is a conserved trait in scleractinian corals and potentially in anthozoans in general. Due to the ubiquity of the polyp bailout, two potential advantages come to mind:

### 4.1 Increased chances of genet survival through polyp bailout

(1) Polyp bailout permits polyps in a modular colony to escape local detrimental factors (e.g., shortage of resources, poor‐quality microhabitats, environmental stress, or pathogens) and hence decreases the risk of mortality of the genets (Acosta et al., 2005; Cook, 1979; Gavish et al., 2018; Hunter, 1984; Rakka et al., 2019; Wells & Tonra, 2021). Furthermore, Gavish et al. (2018) described polyp bailout during *Vibrio coralliilyticus* infection and stated that polyp bailout may provide a coral with an additional defense mechanism enabling the colony to quarantine diseases by “sacrificing” infected polyps. The authors stated that polyp bailout promotes the survival of the genotype by salvaging individual polyps from doomed colonies that may settle and regenerate into new colonies. Under laboratory conditions, lack of food induced bailout, suggesting that the onset of the genetic cascade leading to the stress response can be triggered by a shortage of resources (Goreau & Goreau, 1959; Rakka et al., 2019).

Taken together, these numerous examples show that, due to colony break‐up, the genet is potentially able to avoid stressors by relocating many fragments (ramets). Even though the chances of survival of individual ramets may be low, the number of modules increases the chances of survival of the genet since only a small fraction of the modules need to survive (Acosta et al., 2005; Cook, 1979; Stoner, 1989). Although most studies on scleractinian corals point to problems in resource allocation and successively factors such as growth or regeneration potential of the new ramets (Doropoulos et al., 2012; Edmunds & Elahi, 2007; Hughes & Jackson, 1980), the results obtained, however, have demonstrated that resettlement and survival of the genet itself were successful. Considering the increased mobility of the individual polyps compared to fission‐ or fragmentation‐derived ramets, theoretically one can assume that this survival potential of the genet is increased by polyp bailout due to the modular design of the organism.

### 4.2 Polyp bailout, also a matter of the holobiont?

Another advantageous aspect of polyp bailout could be that the detached polyps retain their microbial symbionts. A microscopic view of the polyps shows that, for example, *Cladocopium* is still present in the cells. This is particularly interesting as there is a clear distinction from coral bleaching, where the algae can be expelled by the coral host (Chuang & Mitarai, 2020; Kvitt et al., 2011; Weis, 2008). Furthermore, there is the theoretical possibility that the microbiome, and thus the established holobiont, will remain in its entirety. In this way, the polyp may be equipped for both direct settlement competence and potential long‐term dispersal. The holobiontic nature of detached polyps raises the question to what extent interkingdom communication supports or even influences polyp bailout. Theoretically, it is conceivable that main drivers of polyp bailout are symbionts or microbiomes. The involvement of a microbe‐associated trigger of polyp bailout has already been proposed (Chuang & Mitarai, 2020), and changes in abiotic as well as biotic factors that can alter the composition of the holobiont also lead to changes in chemical signals delivered by microbes to the coral (Littman et al., 2011; Overstreet & Lotz, 2016; Sharp & Ritchie, 2012; Webster et al., 2013). In this context, one can speak of interkingdom communication between microbes and their eukaryotic hosts (Hughes & Sperandio, 2008; Pacheco & Sperandio, 2009; Segovia, 2008). It has been shown several times that heat stress in combination with the coral pathogen *Vibrio coralliilyticus* can lead to tissue lysis (Ben‐Haim et al., 2003; Vidal‐Dupiol et al., 2011, 2014), which resembles the dissolution of the coenosarc during bailout and resulted in an actual triggering of a polyp bailout response (Gavish et al., 2018). This could function via receptors that recognize “microbe‐associated molecular patterns” (MAMPs) (Yoneyama et al., 2004) and “danger‐associated molecular patterns” (DAMPs) and subsequently trigger caspase‐related apoptotic processes (Newton & Dixit, 2012; Park et al., 2007) as well as regulation of cell survival via the NF‐κB pathway (Williams et al., 2018). Similar patterns have been previously described for polyp bailout (Chuang & Mitarai, 2020; Kvitt et al., 2015; Wecker et al., 2018).

### 4.3 Polyp bailout, a risk‐spreading strategy?

Due to the dispersal capacity of polyps after bailout, this stress response can be compared to a risk‐spreading strategy. Some polyps may sink immediately, whereas others may float away with stronger currents. Since polyp bailout is not necessarily only a clean separation of individual polyps, but sometimes conglomerates of several polyps detach simultaneously (Shapiro et al., 2016; see Figure 1), different dispersal rates can occur under natural flow conditions. A trade‐off may exist among those different types of ramets between size and dispersal, as has been described in fission‐derived conglomerates (patches) of sponges and zoantharians. Larger ramets may sink faster, but their survival time might be longer due to a larger pool of resources (Acosta et al., 2005; Wulff, 1995).

Even small distances (microhabitat changes as shaded vs. nonshaded areas) can improve conditions to such an extent that the polyps can resettle, as stressors are often not uniformly pronounced on the whole reef system (Oliver & Palumbi, 2011), leading to localized survival of the genet (Burgess et al., 2016). On the other hand, single‐detached polyps are small and lightweight and the viscosity and density of seawater and currents alone ensure that the detached polyps are dispersed. Additionally, the ability to survive up to three weeks in the detached state opens up possible new habitats with better conditions and has a positive impact on the selection of this trait and the dispersal of certain genets. Shapiro et al. (2016) reported settlement of detached polyps after as little as 12 hr, with resumed calcification after 2 to 4 days. However, other researchers reported survival of detached polyps without attachment up to 3 weeks (Chuang et al., 2021; Ritchie et al., 1997), implying the possibility that despite the negative buoyancy, polyps will survive long enough to drift away in the water current and colonize more distant undisturbed habitats. Similar observations have been made for brooded *P. damicornis* larvae. These larvae have the competency to immediately settle upon release; nonetheless, they can be dispersed over great distances and stay competent to settle for up to 100 days (Harii et al., 2002; Richmond, 1987).

### 4.4 Potential impacts of polyp bailout for genetic population structure

(2) Polyp bailout may increase fitness by producing many ramets of the identical genetic lineage, capable of survival, and reproduction (Hageman, 2003; Hughes, 1989; McFadden, 1991). The detached polyps can therefore act in the first instance as a mobile stage meeting the basic requirements of brooded larval stages, including immediate settlement competency. While asexual reproduction may not change the susceptibility of modules to mortality during acute stress events, the increased number may increase survival of the genetic lineage (Lasker & Coffroth, 1999). The ecological impact of polyp bailout is controlled by the size of the ramets, the number of ramets resulting from a genet, and the number of genets in a population. For example, splitting into ramets certainly increases the chances of survival of genets in acute periods of stress that would otherwise destroy most or the entire coral colony.

A survival rate of 5%, as described by Sammarco (1982), should have a significant impact on the gene pool of a coral population. Coral colonies have been shown to reach sizes over 5 m in diameter (*Porites* spp., Potts et al., 1985; Coward et al., 2020) and up to 10^6^ polyps (*Plexaura flexuosa*, Beiring & Lasker, 2000). Since polyp bailout exploits the modularity of the colony by dividing a colonial organism into thousands of independent, genetically identical individuals, each with the potential to develop into a new colony, an impact on the gene pool of a coral population can be expected by preserving the existing genetic variations.

Polyp bailout could thereby play an important and yet overlooked process to keep existing genetic variation during short‐term, extreme disturbances. Additionally, the possibility to be directly competent to resettle in suitable microhabitats in the vicinity but also the survival of polyps in a mobile, unattached state with dispersal capability for at least up to several weeks increases the chance of survival and reduces the risk of extinction for the genet (Denno & Roderick, 1992; Ronce & Kirkpatrick, 2001; van Valen, 1971). This could increase gene flow among neighboring reefs or even more distant reefs following disturbances. Assuming a comparable dispersal potential by drift as described for planula larvae, polyp bailout could influence gene flow among neighboring reefs. As polyp bailout could therefore influence the dynamics and persistence of populations, the introduction of allelic variation in populations, species abundance, and distribution, as well as the structure of communities (Dieckmann & Doebeli, 1999; Hanski, 2001; Mouquet et al., 2002), it is paramount to learn more about the evolutionary and ecological consequences and their positive or negative implications for the diversity in natural systems.

### 4.5 Can polyp bailout affect the genetic structure of natural reefs?

Whether polyp bailout can protect diversity and may also increase gene flow needs to be tested in future studies. Currently, we can only examine indirect signs of asexual reproductive processes in reef habitats, which could potentially include polyp bailout. For example, reduced levels of genetic diversity could be expected after stress events.

Sammarco (1982) described *S. hystrix* colonies that showed signs of polyp bailout at Davies Reef in the central region of the Great Barrier Reef. Although neither the cause nor a possible stressor could be identified, and no follow‐up studies on the dispersal or survival rates were conducted, the authors suggested that polyp recovery may be a factor contributing to the dominance of *S. hystrix* in certain shallow reef areas. Similar observations were made by Wild et al. (2014) with *P. damicornis* colonies in Costa Rica. Furthermore, it has been shown that local populations of *Acropora* sp. and *Pocillopora* sp., both able to undergo polyp bailout, can be highly structured by asexual processes (Baums et al., 2006; Drury et al., 2019; Highsmith, 1982; Stoddart, 1984). A study on One Tree Island in the Great Barrier Reef showed high levels of asexual recruitment for certain spots that experienced disturbance events in preceding years (Sherman et al., 2006), while a study prior to these disturbances at this site showed high genotypic diversity and little evidence of asexual recruitment (Benzie et al., 1995). A population of *Pocillopora* colonies at Isabela Island, Galápagos, was reported to be monogenotypic, thus the colonies derived from asexual processes. Recurrent El Niño–Southern Oscillation (ENSO) warming events have caused extensive mortality of reef‐building corals and altered colony size distribution in the Galápagos Islands, suggesting that the colonies regrew from survivors of the 1997/98 ENSO (Baums et al., 2014). Another example is a study on the reefs on Moorea, French Polynesia, which even showed increased recruitment; it cannot be ruled out that this stemmed from asexual processes like polyp bailout (Edmunds, 2017). The authors described a higher number of recruits after stress events compared to periods without stress and during the lack of sexual reproduction period. Similar patterns have been observed in Thailand. A high level of clonality was found for *P. acuta* on the reefs of Koh Phangan, Thailand. *P. acuta* colonies collected for experiments on polyp bailout showed low genotypic diversity and high levels of clonality for certain locations (Gösser, 2019, unpublished data).

Whether all of these observations are due to increased sexual reproduction, other modes of asexual reproduction, or maybe a stress response like polyp bailout that creates a lot of potential new ramets cannot be fully answered here. It should not be ruled out that polyp bailout plays a role in shaping the genetic structure of local reefs after stress events, which also implies that surviving recruits after severe stress periods may not be better adapted to future stress events, but may be just as vulnerable as the previous coral community.

## 5 USE OF POLYP BAILOUT IN MICROSCALE MODEL SYSTEMS

The modular structure and shared life‐history traits of sessile colonial invertebrates, comparable to higher plants, offer a chance to establish model systems for experimental work. Species whose modules are easily propagated provide powerful models for experimental investigations. An approach to create these micropropagates via the break‐up of modularity could be polyp bailout, as demonstrated by Shapiro et al. (2016).

So far in cnidarians, valuable insights into physiological processes such as calcification and symbiosis have relied mostly on the use of cell or tissue cultures (Domart‐Coulon et al., 2001; Feuillassier et al., 2014; Frank et al., 1994; Helman et al., 2008; Lecointe et al., 2013; Mass et al., 2012, 2017). However, the cell dissociation necessary for the production of cell or tissue cultures inevitably leads to the loss of information about tissue spatial organization and alters the physical and chemical conditions in the observed cells. Since it has not yet been possible to produce long‐lived coral cell or tissue cultures (Domart‐Coulon et al., 2001; Helman et al., 2008; Lecointe et al., 2013; Mass et al., 2012), it can be assumed that cells or tissues in such cultures show a deteriorating health status. This suggests that the cells in such cultures are likely to be under stress and therefore represent physiological and metabolic processes that deviate from normal. These potential disadvantages can be compensated for by microscale model systems that function via micropropagation, that is, by the production of tissue explants that are able to develop into whole organisms again (Shapiro et al., 2016; Smith & Drew, 1990; Vizel et al., 2011).

### 5.1 Examples of microscale model systems

Plant science contains numerous examples of such model systems. There, they have been routinely used for the comparative analysis of tissues or whole organisms, resulting in important discoveries for crop production, conservation, and restoration projects (George et al., 2008; Kyte et al., 2013; Smith & Drew, 1990). Such systems have also been developed in the field of cnidarians. In particular, systems of the freshwater polyp *Hydra* sp. have already produced numerous findings regarding the regeneration of injuries and the development of body symmetry and growth (Bosch et al., 2010; Gierer, 2012; Guder et al., 2006). Microscale model systems have also been developed for the sea anemones *Aiptasia* sp. (Rädecker et al., 2018; Schlesinger et al., 2010) and *Nematostella* sp. (Darling et al., 2005; Rabinowitz et al., 2016), as well as for the upside‐down jellyfish *Cassiopea* sp. (Cabrales‐Arellano et al., 2017; Ohdera et al., 2018).

Micropropagation enables the rapid production of tens to hundreds of genetically identical replicates, which could form the basis for ecological model systems by selecting known genotypes so that genotype‐dependent responses can be neglected or tested for in experiments. In addition, cells and tissues can be viewed in their original physiological state without impairment, since micropropagation preserves much of the complexity of the coral. This opens up research on symbioses between the coral host, the zooxanthellae, and the associated bacterial community (Chuang & Mitarai, 2020; Fordyce et al., 2017).

In Scleractinia, the solitary scleractinian coral *Fungia granulosa* has been in the focus of development of a microscale‐ecological model system (Gardner et al., 2015; Vizel et al., 2011). Their advantage is their high ability to regenerate their tissue and to use a process called anthocauli budding under detrimental conditions like environmental stress, predation, or partial burial under sediment, which allows tissue explants to regenerate (Kramarsky‐Winter & Loya, 1996). In the microscale system developed, tissue fragments of certain sizes have been shown to develop into planula‐like spheres that settle and grow into new, fully differentiated individual corals (Gardner et al., 2015; Vizel et al., 2011). Furthermore, for scleractinian corals, tissue explants and cells have been shown to aggregate into ball‐like spheres called “proto‐polyps” or “tissue balls” (Domart‐Coulon et al., 2004; Feuillassier et al., 2014; Mass et al., 2012). These structures have been used for studies on biomineralization and symbiosis, but have the same problem as cell and tissue cultures as they only stay viable for days to weeks. To date, it could not be shown that these cell aggregates can regenerate into a whole individual coral colony again. Therefore, using polyp bailout to produce micropropagates that still retain their original organization could be a useful tool for the development of microscale model systems for Scleractinia.

### 5.2 Application of polyp bailout for micropropagation

**FIGURE 2:**
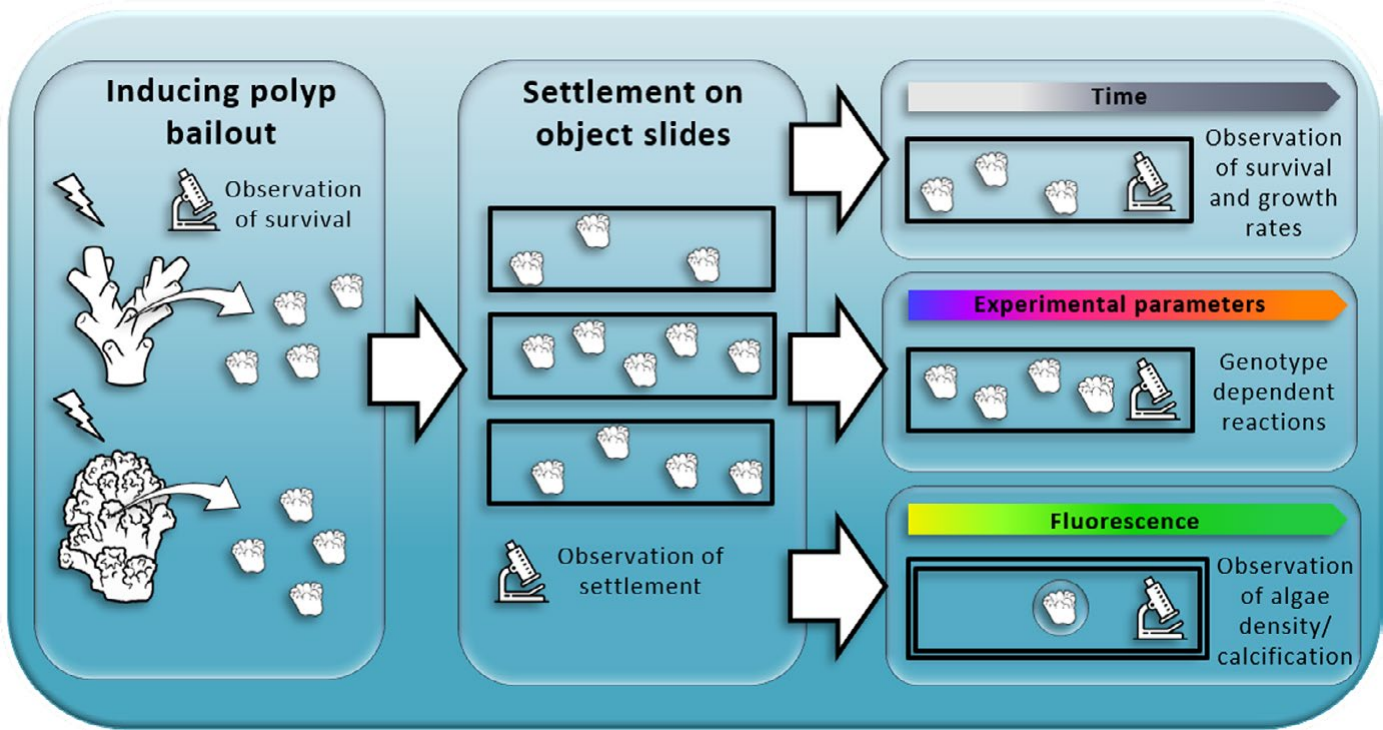
Schematic workflow of polyp bailout in a microscale model system in future studies. Induction of polyp bailout and verification of polyp vitality. Settlement of the polyps on suitable material, for example, microscope slides. After settlement, general monitoring of ecological parameters over specific time periods. Use in experiments with high number of replicates and possibility to study genotype‐dependent differences. Monitoring the condition of the polyps with special microscopy techniques, such as fluorescence microscopy

The coral‐on‐a‐chip microfluidic platform reported by Shapiro et al. (2016) shows the feasibility of such a microscale model system for Scleractinia. Coral fragments from three different coral species, all from the pocilloporid family, were used to generate the micropropagates. Increasing levels of salinity induced the onset of polyp bailout. After the bailout, viable detached polyps were placed on microscope slides with microwells and transferred to a raceway system with constant laminar water flow. Settled polyps on microscope slides could then be placed in a microfluidic system that allowed microscopic observation and measurement of fundamental behaviors and physiological processes in vivo. Advantages over traditional macroscale systems are the small amount of coral tissue needed, temporal resolution, and small space required. Liu et al. (2020) also used salinity as the trigger of polyp bailout to produce micropropagates. Detached polyps were fixed shortly after initiation of reattachment and examined for various aspects using a combination of tissue clearing and fluorescence microscopy with an immunofluorescence assay. A very similar system to Shapiro et al. (2016), using polyp bailout as the source of micropropagation and keeping the detached polyps in a microfluidic system without prior settlement, was recently described by Luo et al. (2020). In contrast to Shapiro, however, salt stress was not used as a trigger for polyp bailout in *Pocillopora* fragments, but rather calcium‐free seawater, a technique that has been used previously to obtain cell and tissue cultures in corals (Domart‐Coulon et al., 2001; Frank et al., 1994; Muscatine et al., 1998). Furthermore, no settlement of the polyps was described here; instead, the detached polyps were transferred directly to a microfluidic system where they could be kept alive for two weeks. This duration does not make any difference to the cell and tissue cultures or "tissue balls" described so far. Even though it could be shown that the developed microfluidic system prolonged the lifespan of the free polyps (Pang et al., 2020), a deteriorating health status of the polyps must be assumed, which could falsify observations in subsequent experimental setups. Nonetheless, polyp bailout seems to be a promising tool for the design of microscale‐ecological model systems (see Figure 2). The small required space, temporal resolution, high reproducibility, and generation of a high number of genetically identical replicates are benefits over traditional experimental setups in coral research.

## 6 FUTURE RESEARCH

**FIGURE 3:**
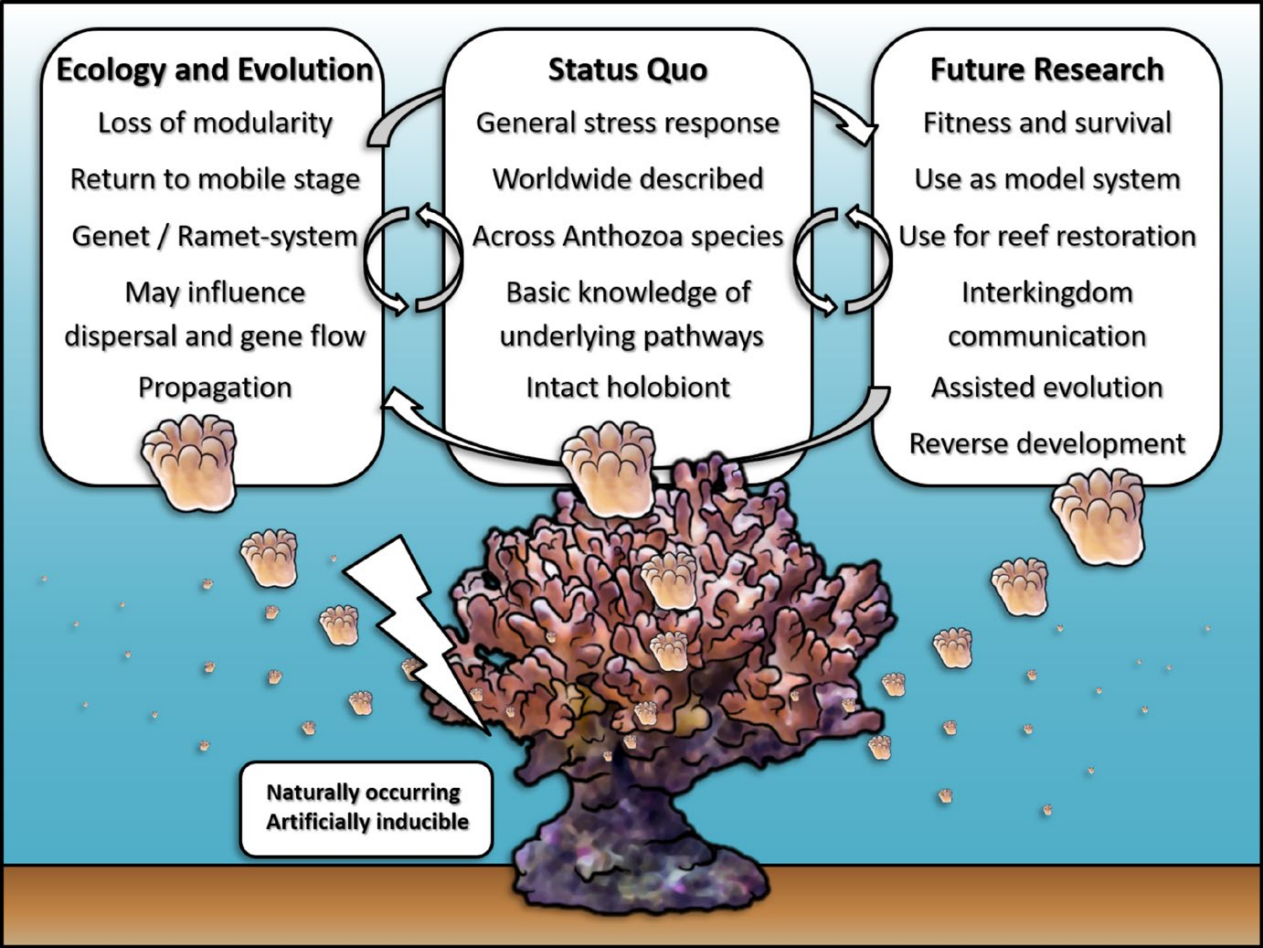
Summary of polyp bailout related knowledge. Based on the current state of knowledge about the stress response polyp bailout, first considerations regarding ecological and evolutionary relevance as well as further research questions arise

Although the topic of the polyp bailout has become relevant, many questions regarding the stress response are still unresolved. Previous studies have dealt with the factors leading to polyp bailout or its implications for the use in microscale‐ecological model systems. Therefore, future work should take into account different research topics (see Figure 3). Several topics seem promising for future research.

First, the regulatory processes underlying polyp bailout need to be investigated in more detail, both with more species and with more stressors. Only comparable studies including a broad variety of scleractinian corals or cnidarians in general, such as the hydrozoan *Millepora* sp., and exposure to different stress regimes, will provide a detailed overview of species and stressors.

Second, modern approaches, as recently published by Chuang and Mitarai (2020), using RNAseq to identify the gene regulatory pathway underlying the different stages of the stress response should be used. In particular, the stages following colony detachment from freshly detached polyps up to surviving polyps or even polyps during resettlement need to be investigated on the physiological as well as molecular regulatory level as commenced by Chuang et al. (2021) for *P. acuta*. Little work has been done addressing the stages following polyp bailout. Renewed skeletogenesis of resettled polyps (Sammarco, 1982; Shapiro et al., 2016) and normalization of gene expression of detached polyps to a state prior to the onset of polyp bailout have been shown (Chuang et al., 2021), but further observations of ongoing growth and long‐term survival are missing. Understanding the regulatory processes of all stages during and after polyp bailout will allow distinction of polyp bailout from other stress responses such as coral bleaching (Chuang et al., 2021; Chuang & Mitarai, 2020) and may provide knowledge about stress responses in general, but also their delimitation into triggers and responses in particular. Hence, it is necessary to investigate which corals species can undergo polyp bailout, what threshold exists to trigger polyp bailout, and whether the noncoral parts of the holobiont may be involved in the stress response. For that purpose, interkingdom communication at the molecular level should provide a promising approach to find out the extent to which the existing microbiota and/or algae exert regulatory influences on polyp bailout (Chuang et al., 2021; Chuang & Mitarai, 2020; Gavish et al., 2018).

### 6.1 Unresolved questions regarding polyp bailout

Until now, few studies have tried to resettle the detached polyps, with varying degree of success and almost exclusively conducted in the laboratory (Liu et al., 2020; Luo et al., 2020; Pang et al., 2020; Shapiro et al., 2016). Hence, the proof or at least evidence that survival and resettling take place under natural conditions is still lacking, and survival/resettlement rates and dispersal competence in natural reef systems are unknown for polyp bailout. Future studies of colonization and long‐term survival rates of individual polyps must therefore be carried out under both laboratory and natural conditions on the coral reefs. Only with this type of fieldwork can we ask: Does polyp bailout lead to the establishment of new colonies? Can polyp bailout maintain genetic diversity during periods of stress? Does this only apply to exceptional species like *P. acuta*, or to different reef builders? If polyp bailout does not change the susceptibility of corals to acute stress events, will it lead to mortality in more severe stress periods? Are colonies that arose from polyp bailout more resilient through “epigenetic” effects or adaptive responses, as has been already indicated in some corals (Dixon et al., 2016, 2017; Putnam et al., 2016)? Is there a possibility that polyps can drift away and find refuge in locally distant or deeper mesophotic zones? Does polyp bailout lead to higher gene flow? The more we know about the physiological, gene regulatory, and ecological processes, and thus the requirements of the detached polyps, the better we can evaluate whether polyp bailout is potentially a useful tool for reef restoration.

### 6.2 Polyp bailout—a tool for reef restoration?

In order to assess the use of polyp bailout as a tool for reef restoration, several gaps in our knowledge must be filled. Little is known about the survival rates of detached polyps and results differ significantly between studies. Sammarco (1982) describes settlement rates with calcification of 4.8% after 9 days, while Shapiro et al. (2016) describe settlement rates of up to 90% in some experiments. Chuang et al. (2021) report 82% vital polyps after 5 days, of which only 52% attain normal polyp morphology but no settlement. Furthermore, as polyp bailout is often described incidentally, replicable protocols for polyp bailout in specific species are lacking. To date, only instructions based on salinity stress in pocilloporids that resulted in replicable induction of polyp bailout have been described (Chuang et al., 2021; Chuang & Mitarai, 2020; Liu et al., 2020; Shapiro et al., 2016). A comparison with techniques used as tools for reef restoration, such as fragment‐oriented approaches (Bowden‐Kerby, 2008; Forsman et al., 2015; Page et al., 2018; Rinkevich, 2014) or sexually produced recruits (Edwards & Gomez, 2007; Guest et al., 2014; Heyward et al., 2002; Nakamura et al., 2011; Omori, 2019), will only be possible when manuals for more coral species, as well as more data on survival rates, settlement rates, and growth rates, become available.

Nevertheless, polyp bailout possesses the potential to produce large numbers of corals from a single coral colony. Thus, new individuals could be continuously grown from parent corals, drastically reducing the need to harvest wild corals from coral reefs or wait for annual or cyclical spawning events to harvest larvae. Additionally, as part of assisted evolution, it is conceivable to generate artificial chimeras from two or more detached polyps that may be better adapted to future conditions, similar to previously described approaches being pursued with coral larvae (Amar et al., 2008; Rinkevich, 2019).

## 7 CONCLUSION

Polyp bailout might provide a platform for highly replicable ecological and evolutionary experiments. By using polyp bailout as a starting procedure, it may be possible to redo a single experiment with tens to hundreds of clones. Thus, polyp bailout is not only a highly interesting stress response, but potentially a tool that might revolutionize coral experiments by providing fast responses and high replicability that are most likely more space and cost‐efficient relative to tank experiments.

## CONFLICT OF INTEREST

None declared.

## AUTHOR CONTRIBUTIONS

**Maximilian Schweinsberg:** Conceptualization (equal); Data curation (equal); Formal analysis (equal); Funding acquisition (supporting); Investigation (lead); Methodology (equal); Project administration (equal); Visualization (equal); Writing‐original draft (equal); Writing‐review & editing (equal). **Fabian Gösser:** Conceptualization (equal); Data curation (equal); Formal analysis (supporting); Funding acquisition (supporting); Investigation (equal); Methodology (equal); Visualization (equal); Writing‐original draft (equal); Writing‐review & editing (equal). **Ralph Tollrian:** Conceptualization (equal); Data curation (supporting); Formal analysis (supporting); Funding acquisition (equal); Investigation (supporting); Methodology (equal); Project administration (equal); Writing‐original draft (supporting); Writing‐review & editing (equal).

## Data Availability

No data were used.
